# Effects of Acute and Chronic Exposure to Residual Level Erythromycin on Human Intestinal Epithelium Cell Permeability and Cytotoxicity

**DOI:** 10.3390/microorganisms7090325

**Published:** 2019-09-06

**Authors:** Haihong Hao, Kuppan Gokulan, Silvia A. Piñeiro, Katherine M. Williams, Zonghui Yuan, Carl E. Cerniglia, Sangeeta Khare

**Affiliations:** 1 Division of Microbiology, National Center for Toxicological Research, US Food and Drug Administration, 3900 NCTR Rd, Jefferson, AR 72079, USA; 2 Division of Human Food Safety, Center for Veterinary Medicine, US Food and Drug Administration, 7500 Standish Place, Rockville, MD 20885, USA; 3 National Reference Laboratory of Veterinary Drug Residues (HZAU), College of Veterinary Medicine, Huazhong Agricultural University, Wuhan 430070, China

**Keywords:** erythromycin, epithelial cell barrier function, gene expression, acute or multiple dose exposure, permeability, single dose exposure

## Abstract

Residual concentrations of erythromycin in food could result in gastrointestinal tract exposure that potentially poses a health-hazard to the consumer, affecting intestinal epithelial permeability, barrier function, microbiota composition, and antimicrobial resistance. We investigated the effects of erythromycin after acute (48 h single treatment with 0.03 μg/mL to 300 μg/mL) or chronic (repeated treatment with 0.3 µg/mL and 300 µg/mL erythromycin for five days) exposures on the permeability of human colonic epithelial cells, a model that mimics a susceptible intestinal surface devoid of commensal microbiota. Transepithelial electrical resistance (TER) measurements indicated that erythromycin above 0.3 µg/mL may compromise the epithelial barrier. Acute exposure increased cytotoxicity, while chronic exposure decreased the cytotoxicity. Quantitative PCR analysis revealed that only *ICAM1* (intercellular adhesion molecule 1) was up-regulated during 0.3 μg/mL acute-exposure, while *ICAM1*, *JAM3* (junctional adhesion molecule 3), and *ITGA8* (integrin alpha 8), were over-expressed in the 300 μg/mL acute treatment group. However, during chronic exposure, no change in the mRNA expression was observed at 0.3 μg/mL, and only *ICAM2* was significantly up-regulated after 300 μg/mL. *ICAM1* and *ICAM2* are known to be involved in the formation of extracellular matrices. These gene expression changes may be related to the immunoregulatory activity of erythromycin, or a compensatory mechanism of the epithelial cells to overcome the distress caused by erythromycin due to increased permeability.

## 1. Introduction

Erythromycin, a member of the macrolide antimicrobials, has been widely used in humans and animals for the treatment of a number of bacterial infections for Gram-positive cocci and Gram-negative cocci, besides mycoplasma infections [1,2]. Erythromycin exhibits antimicrobial as well as immunomodulatory properties [3,4]. Pharmacological studies have shown that 37–43% of orally administered erythromycin could remain in the gastrointestinal tract (GIT) [5,6]. Erythromycin A and its closely related semisynthetic molecules rank among the top three antimicrobial agents used in food producing animals. In fact, erythromycin A is a commonly used medicine in poultry industry to treat chronic infections due to mycoplasma [7]. Antimicrobials, including erythromycin residues, have been detected in animal feedstuffs such as spent distillery grains from ethanol production [8]. Furthermore, the therapeutic use of erythromycins in veterinary medicine as injectables, feed additives, or in drinking water to treat, control, and prevent disease in food animals could lead to its presence as a residue in food. If withdrawal times for clearance of this antimicrobial are not strictly followed, excessive exposure to erythromycin and its metabolites to the GIT can occur, raising questions about potential effects on human health [9,10]. The PK/PD study revealed that erythromycin is metabolized into anhydroerythromycin (AHE) by gastric fluids [11]. The hydrolysis mechanism is more effective in acidic conditions compared to neutral or basic conditions. Erythromycin and AHE are differentially distributed in blood, tissue, and leukocytes during and following therapy [11]. A detectable concentration of erythromycin was present in these sites/cells 72 h after the treatment; however, the proportion of AHE becomes higher after the end of treatment. Erythromycin is metabolized in the liver through a *N*-demethylation process, resulting in des-*N*-methyl-erythromycin. AHE and other erythromycin metabolites are known to be microbiologically inactive; however, an in vitro study showed that AHE inhibits the oxidation function of Cytochrome P450 more efficiently than the erythromycin itself [12]. This inhibitory function may decrease drug clearance in the tissue, resulting in tissue accumulation, and be a mechanism for drug-induced toxicity.

Des-*N*-methyl-erythromycin is not excreted through urine; rather, this metabolite is absorbed by the GIT [13,14]. Moreover, the majority of the erythromycin metabolites are recovered from the GIT and feces [10]. The presence of commonly used veterinary drugs and their metabolic products in the intestinal tract may result in permeability changes in intestinal epithelial cells [15,16]. Intestinal permeability is closely related to inflammatory conditions and the invasion of pathogenic microorganisms [17,18,19,20]. While assessing the toxicity of erythromycin, the Joint FAO/WHO Expert Committee of Food Additives (JECFA) considered that microbiological effects, specifically disruption of the colonization barrier, were more relevant than toxicological effects for the establishment of an acceptable daily intake (ADI) for erythromycin.

Intestinal microbial populations maintain gastrointestinal homeostasis and limit epithelial permeability [21]. Current evidence suggests that increased permeability of the gut due to perturbation of the intestinal microbiota by xenobiotics may prompt cycles of intestinal inflammation and could be linked to many conditions, including Crohn’s disease, ulcerative colitis, Type I diabetes, irritable bowel syndrome, GIT infections, celiac disease, and graft-versus-host disease [17,22]. Intestinal permeability is mainly regulated by proteins associated with epithelial cell junctions. These proteins can link epithelial cells together into a functional barrier, capable of blocking the passage of microorganisms and larger molecules while allowing absorption of water and nutrients [17,18].

The purpose of this study was to assess the in vitro effects of residual concentrations of erythromycin on the intestinal epithelial layer using T84 cells, a human colorectal carcinoma cell line. We investigated using a comprehensive methodological approach that included transepithelial electrical resistance (TER), cytotoxicity, and mRNA expression levels to determine the key genes involved in intestinal integrity after exposure to erythromycin. To our knowledge, this is the first study using the T84 cell line to evaluate the potential effects of residual amounts of erythromycin on human intestinal epithelial cells. In this research, monolayers of T84 cells were incubated with a range of erythromycin concentrations (0.03 μg/mL to 300 μg/mL) that included a residual dose (0.3 μg/mL) and a high dose (300 μg/mL) [23] to evaluate changes in paracellular permeability and cell toxicity.

## 2. Material and Method

### 2.1. Cell Culture

T84 cells (ATCC CCL-248™), a human colorectal carcinoma cell line, were obtained from ATCC (Manassas, VA, USA). Complete growth media was composed of Dulbecco’s Modified Eagle Medium (DMEM)/F-12 media with L-glutamine and HEPES (both ATCC), and supplemented with 5% fetal bovine serum (FBS), fungizone, and penicillin/streptomycin as described earlier [16]. Cells were grown in complete media using 75 cm^2^ cell culture flasks until approximately 70–80% confluent and then split using 0.25% Trypsin–EDTA into desired vessels. Cell cultures were maintained at 37 °C with 5% CO_2_ and 95% humidity.

### 2.2. Transwell Model and Transepithelial Electrical Resistance (TER) Measurement

T84 cells were seeded onto 6.5 mm collagen-coated transwell inserts (Corning, NY, USA) at a concentration of 2.0 × 10^5^ cells/well. Cell monolayers were grown for 5–10 days. Once TER of cell monolayers reached approximately 800–1000 Ω, antimicrobial free media was exchanged twice to apical and basal reservoirs. Measurement of TER was performed according to methods established previously [16,24,25,26,27]. TER readings were taken using a STX electrode probe and EVOM2 Epithelial Voltammeter (World Precision Instruments, Sarasota, FL, USA). Once TER of polarized cell monolayers again reached approximately 800–1000 Ω, media was changed, and cells were equilibrated for 3 h. Baseline TER control readings were taken before starting the acute or chronic exposure to erythromycin.

### 2.3. Acute Exposure of Erythromycin

Erythromycin was dissolved in ethanol; and the final concentration of ethanol in the treatment media was below 1.5%. Cells treated with media containing 1.5% ethanol served as control. Polarized cells were exposed to single dose of erythromycin (0.03 μg/mL to 300 μg/mL) for 48 h. This dose range included the erythromycin concentration above, below, and at residual concentration exposure levels [16,23]. The JECFA established an ADI of 0–0.7 µg/kg bw erythromycin concentration, based on microbiological effects, as more relevant concentration/dose used for risk assessment with microbiota than the toxicological endpoints [6]. Based on the TER results (as described below), subsequent experiments were performed with only 0.3 and 300 μg/mL. To assess changes in epithelial cells resistance, TER readings were taken at 0, 1, 3, 24, and 48 h post-exposure. TER measurements are presented as fold of original baseline value (measured before addition of erythromycin or controls) and statistical data analysis was performed using Sigma Plot (Systat Software, San Jose, CA, USA).

### 2.4. Chronic Exposure of Erythromycin

For the chronic treatment, cell monolayers were treated with 0, 0.3, and 300 μg/mL erythromycin daily for 5 days. Medium was replaced with fresh medium containing erythromycin (0, 0.3, and 300 μg/mL) each day. Drug treatment was performed after 3 h equilibration (to avoid any fluctuation due to the change in the media) and TER measurements were taken at 0, 1, 3, 20, and 22 h post-exposure every day. TER measurements and statistical data analysis was performed the same way as with single treatment.

### 2.5. Cytotoxicity Assay

Supernatants from the apical and basal compartments of each transwell were collected to assess the cell cytotoxicity. The CytoTox 96^®^ Non-Radioactive Cytotoxicity Assay was performed using the manufacturer’s instructions (Promega Corporation, Madison, WI, USA). This assay measures lactate dehydrogenase (LDH), a stable cytosolic enzyme released upon cell lysis. Released LDH in culture supernatants was measured through a 30-minute coupled enzymatic assay, which results in conversion of a tetrazolium salt (INT) into a red formazan product. The amount of color formed is proportional to the number of lysed cells. Visible wavelength absorbance (OD490) data were collected using a standard 96-well plate reader (Bio-Tek, Winooski, VT, USA).

### 2.6. qPCR Analysis of Genes Involved in the Cellular Integrity

mRNA was extracted from the cells attached to the transwell membrane using the PARIS kit (Life Technologies, Carlsbad, CA, USA) according to the manufacturer’s instructions. The concentration of RNA was quantified using a NanoDrop^®^ ND-1000 (NanoDrop, Wilmington, DE, USA). Extracted RNA was treated with the Turbo DNA-free kit (Life Technologies, Carlsbad, CA, USA). mRNA was reverse transcribed to make cDNA using the Two-Step Reverse Transcription TaqMan kit (Applied Biosystems, Foster City, CA, USA). Gene expression analysis of 84 cell junction-related genes was examined using the RT2 Profiler PCR Array Human Cell Junction Pathway Finder (Qiagen, Valencia, CA, USA). Plates were analyzed using an ABI 7500 Real-Time PCR system (Life Technologies, Carlsbad, CA, USA), with amplification conducted with an initial 10-minutes step at 95 °C followed by 40 cycles of 95 °C for 15 s and 60 °C for 1 min. One independent sample was used for each plate and three plates (mRNA obtained from three independent experiments) were run for each experimental group. Data was analyzed using the web-based Qiagen data analysis tool, and using β-ACT, β2 M, GAPDH, HPRT1, and RPLP0 housekeeping genes. Data from T84 cells treated with 0.3 and 300 μg/mL erythromycin were individually compared to control cells. Changes in gene expression for RT2 Profiler plates were evaluated by Student’s *t* test [28]. Genes with *p* < 0.05 were designated as significant.

## 3. Results

### 3.1. Effect of Acute Exposure on Epithelial Cells Permeability

**Figure 1:**
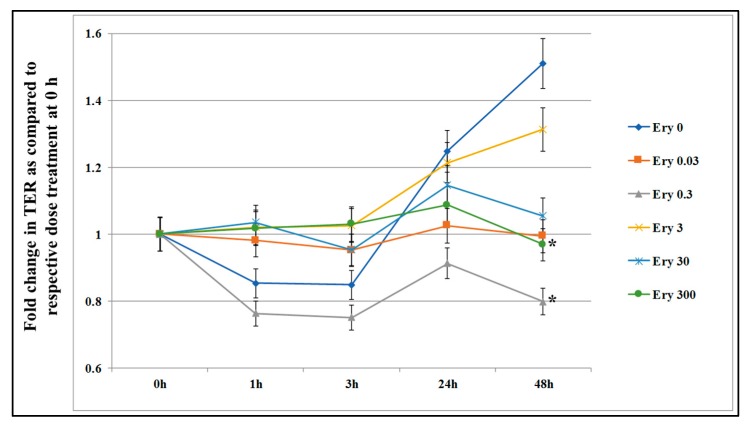
Effect of acute exposure of erythromycin on intestinal epithelial cell permeability. T84 cells were seeded in transwells and culture media was periodically changed until transepithelial electrical resistance (TER) value reached 800-1000 Ω. The TER value was recorded before and after incubation with five concentrations of erythromycin (Ery 0.03 = 0.03 μg/mL; Ery 0.3 = 0.3 μg/mL; Ery 3 = 3 μg/mL; Ery 30 = 30 μg/mL; and Ery 300 = 300 μg/mL) at 0, 1, 3, 24, and 48 h. The TER value recorded before adding erythromycin (time 0) served as the baseline. Data are presented here as fold increase or decrease in the same well before treatment. Each value represents an average of three independent experiments. * indicates the statistical significance (*p* < 0.05).

Figure 1 shows that the untreated control cells revealed a healthy cell status as the TER increased slowly and steadily. At the 24 h time point there was no difference in TER value between control and erythromycin treated cells. The lowest concentration of erythromycin (0.03 μg/mL) did not reveal any changes in the TER during the entire 48 h period. In contrast, at 48 h time point, erythromycin 300 μg/mL and 0.3 μg/mL treated wells showed significant decrease (*p* < 0.05) in TER as compared to control, indicating that the erythromycin may increase permeability of the cell layer. The residue concentration (0.3 μg/mL) and a concentration known to be toxic to cells (300 μg/mL) were selected to assess the effect of single treatment on the expression of mRNA genes related to epithelial permeability.

### 3.2. Effect of Chronic Exposure on Epithelial Permeability

**Figure 2:**
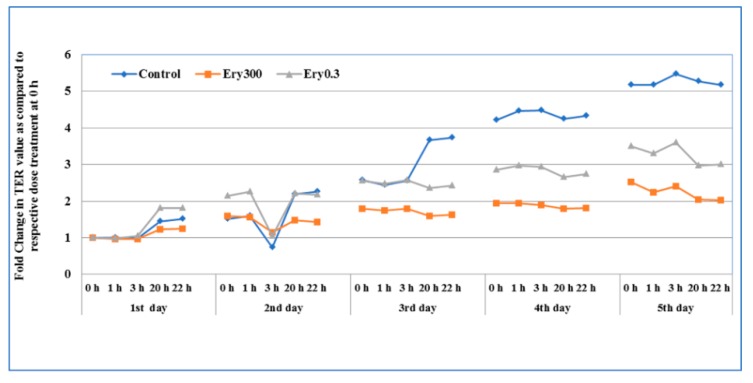
The effect of chronic exposure of erythromycin on the intestinal epithelial cell permeability. T84 cells were seeded in transwells and culture media was periodically changed and cultured until TER value reached 800 to 1000 Ω. The TER value was recorded (at 0, 1, 3, 20, and 22 h) after daily treatment of erythromycin at two concentrations (E0.3 = 0.3 μg/mL and E300 = 300 μg/mL). Data here represent the fold change after daily treatment at each time point (*n* = 3) for five days. Erythromycin-free media in the control wells was also replaced at the same time.

The TER following chronic erythromycin exposure was tested to assess the changes in the intestinal epithelial cell permeability. For chronic exposure, media of the T84 cells grown on transwells was replaced daily with respective treatment condition (media containing erythromycin 0.3 μg/mL and 300 μg/mL) for five days. After chronic daily exposure to 0.3 and 300 μg/mL doses for five days, the changes in cell layer TER were much more dramatic, with significant increases in permeability observed after two days of exposure (Figure 2). When compared with the control epithelial cells, exposure to erythromycin at both concentrations resulted in >2-fold TER suppression after five days of treatment, indicating long-term treatment with erythromycin could increase permeability. The result showed that erythromycin causes TER suppression from day three onwards. The highest concentration of erythromycin induced the highest level of TER suppression that was statistically significant.

### 3.3. Effect of Acute Erythromycin Exposure on Cell Cytotoxicity

**Figure 3:**
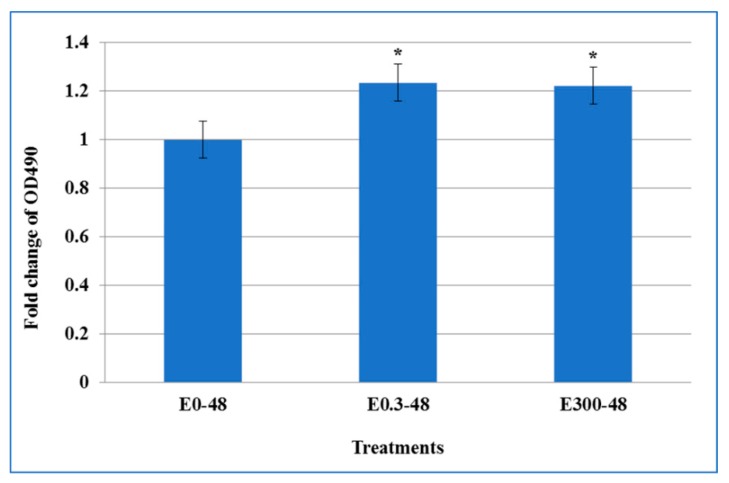
The effect of acute exposure of erythromycin on cell cytotoxicity. Cell culture supernatants from the transwell apical compartment were assessed to determine the cell cytotoxicity by measuring lactate dehydrogenase (LDH), a stable cytosolic enzyme that is released upon cell lysis. Released LDH in culture supernatants was measured with a 30-minute coupled enzymatic assay, which results in conversion of a tetrazolium salt (INT) into a red formazan product that is quantitated at wavelength absorbance of (OD_490_). Values here depict the fold change in LDH release as a measure of cytotoxicity caused by erythromycin exposure on T84 cells after 48 h single treatment as compared to untreated controls. * indicates the statistical significance (*p* < 0.05).

The culture medium was collected from the basal and apical transwell compartments, and cell cytotoxicity was assessed by measuring the LDH. The results showed no difference in the LDH concentration in the basal compartment between control cells and cells treated with two different concentrations of erythromycin (data not shown). In contrast, as shown in Figure 3, in the apical compartment cell culture supernatants, the released LDH was significantly increased after 48 h single treatment of 0.3 and 300 μg/mL erythromycin (*p* < 0.05), indicating that these two concentrations resulted in more cell lysis than that in the drug free control.

### 3.4. Effect of Chronic Erythromycin Exposure on Cytotoxicity

**Figure 4:**
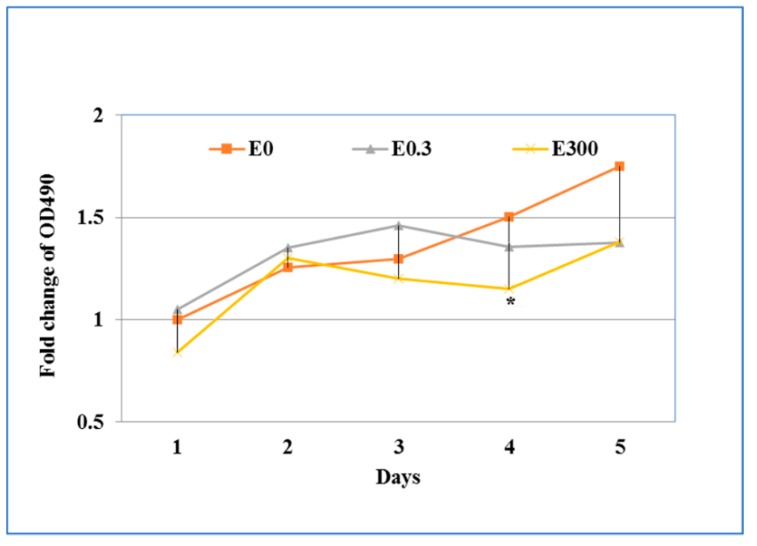
The effect of chronic exposure of erythromycin on cell cytotoxicity. Cell culture supernatants from the apical compartment of transwells were collected each day to determine the cell cytotoxicity by measuring released LDH as described in Figure 3. * indicates the statistical significance (*p* < 0.05).

During the chronic exposure, there was no significant difference of the OD_490_ value in the media collected from the basal suspension (data not shown). Within the erythromycin-free controls, LDH release increased in the culture supernatants collected from the apical compartment over the five day culture interval. The daily exposure of T84 cells to 0.3 μg/mL erythromycin did not result in significant change of LDH during the five days as compared with controls. However, when the cells were treated daily with 300 μg/mL erythromycin, LDH release increased in the first two days but decreased the next two days (Figure 4). Compared to the drug free treatment, significant difference was observed in day four after daily treatment of 300 μg/mL erythromycin (*p* < 0.05).

### 3.5. Impact of Acute Exposure on Expression of Human Cell Junction Genes

T84 epithelial cell monolayers were evaluated for changes in gene expression resulting from exposure to erythromycin. Effects of 48 h exposure to erythromycin (0.3 and 300 μg/mL) were evaluated using a PCR array plate with 84 genes related to cell junctions and permeability. Several genes were differentially regulated during erythromycin treatment (Table 1). Genes with significant changes in expression were those with *p* < 0.05 when compared to control. Overall, the expression of only three genes was significantly changed in both groups (*p* < 0.05). Treatment with 0.3 μg/mL erythromycin only resulted in significant changes (about two-fold) in one gene, intercellular adhesion molecule 1 (*ICAM1*). Following exposure to 300 μg/mL erythromycin, T84 cells demonstrated up-regulation of *ICAM1,* junctional adhesion molecule 3 (*JAM3*), and integrin alpha 8 (*ITGA8*). Of those genes, *ITGA8* showed the greatest changes in expression, with a 175-fold increase. Additionally, exposure to 0.3 μg/mL and 300 μg/mL erythromycin resulted in altered expression of genes encoding caveolin (*CAV1/3*), cadherin (*CDH2*), claudin (*CLDN10/11/14/17/18/19*), desmocollin (*DSC1/3*), gap junction proteins (*GJA1/3/5, GJB4/5/6, GJC3*), integrin (*ITGA4/7, ITGAM*), and notch (*NOTCH4*), with more than two-fold changes that could be biologically significant, but were not significant statistically.

### 3.6. Impact of Chronic Exposure of Erythromycin on the Expression of Human Cell Junction Genes

No genes were significantly changed after five days of treatment by 0.3 μg/mL erythromycin (Table 2). Only one gene, *ICAM2*, was significantly up-regulated (*p* < 0.05) when exposed to 300 μg/mL erythromycin for five days (Table 2). However, the *ICAM2* up-regulation was less than a two fold. The chronic treatment by 0.3 μg/mL and 300 μg/mL erythromycin resulted in different expression of genes encoding caveolin (*CAV3*), claudin *(CLDN8/10/14/17/18/19),* desmocollin *(DSC1),* desmoglein (*DSG3),* gap junction proteins *(GJA5, GJB6, GJC2)*, and integrin *(ITGA4, ITGAM, ITGB3*), with more than a two-fold change. Of those genes, the *CAV3, CLDN14, CLDN17, DSC1, ITGA4*, and *ITGB3* were down-regulated, while the *CLDN18, CLDN19, DSG3, GJA5, GJB6* were up-regulated, after treatment by both 0.3 μg/mL and 300 μg/mL erythromycin. However, while the observed changes might have been biologically significant, they were not significant statistically.

## 4. Discussion

Despite reports of several therapeutic dose-related side effects, including gastrointestinal disturbances, erythromycin is widely used in human and veterinary medicine to combat certain bacterial infections. Risk assessment of an antibiotic is conducted by determining the potential of intestinal barrier disruption with an emphasis on the effect on commensal microbial population and development of resistance against antimicrobial drug. Another aspect of intestinal barrier disruption is the direct effect of residual antibiotic on the integrity of intestinal epithelial cells, especially, as some of the antibiotics stay in the intestine for more than 48 h. One of the most important functions of the gastrointestinal epithelium, and supporting elements such as the mucus layer, defensins and the commensal bacteria residing on the intestinal mucosa, is to function as a physical and biochemical barrier between the luminal contents and the interior of the human body [17,29,30]. As such, epithelial integrity can serve as an indicator of overall gastrointestinal health. Several studies have shown that the bacterial cell wall ultrastructure, cell membrane permeability, morphology, and ability to form bacterial biofilms are affected during antimicrobial treatment [31,32,33,34]. There is a knowledge gap in describing the effects of residual amounts of antimicrobial agents on intestinal epithelial cells. Moreover, our group recently described the effects of acute (single) exposure to residual levels of tetracycline on the barrier functions of human intestinal epithelial cells [16]. The present study is an in vitro approach to assess whether an acute (single) or chronic (repeated for five days) exposure to residual levels of erythromycin has any adverse effects on intestinal epithelial cells.

Acute use of erythromycin was monitored for 48 h after single treatment with erythromycin above, below, and at residual concentrations, whereas, the chronic use of erythromycin was replicated by replacing culture media with fresh media containing erythromycin daily for five days. The acute exposure reflected the interaction of a residual amount of erythromycin as a one-time event, and the chronic exposure reflected the exposure of a residual amount of erythromycin daily for five days. In terms of exposure to consumers ingesting erythromycin it reflects occasional or daily consumption of edible products with residual amounts of erythromycin, respectively.

During acute treatment, a biphasic effect of erythromycin was noticed; at lower concentrations no change (0.03 μg/mL) or a decrease (0.3 μg/mL) in the TER was seen. However, at subsequent concentrations an increase in TER (3 μg/mL) or almost no change in TER (30 and 300 μg/mL) was noticed. These observations could be also correlated with the erythromycin having a biphasic effect on migrating motor complex (MMC—housekeeper of the GIT) cycle on the stomach and small intestine [35]. This biphasic effect of erythromycin on MMC is proposed to be due to its agonist activity towards motilin, a gastroprokinetic hormone [35,36,37]. Another agonist of motilin (mitemcinal) also showed nonlinear pharmacokinetics [36]. It is well known that many drugs (orally administered) are absorbed by intestinal epithelial cells and attain systemic circulation after passage through the liver via portal veins. The membrane permeability in gastrointestinal epithelial cells (first-pass metabolism) contributes to the nonlinear pharmacokinetics in animals and humans [36]. Thus, intestinal epithelial cell model provides an [35] in vitro platform to test the first pass metabolism of orally administered drugs.

An increase in LDH release (cytotoxicity effect) was observed following 48 h exposure to erythromycin concentrations above 0.3 μg/mL. Due to the increased permeability (decreased TER) of the epithelial cells, an erythromycin concentration above 0.3 μg/mL may pass from the apical compartment of the transwell to the basal compartment without providing optimal time for the interaction of the drug with epithelial cells. Thus, subsequent experiments were conducted using 0.3 μg/mL and 300 μg/mL concentrations only. During the chronic treatment, significant increases in the intestinal cell epithelium permeability were observed and were accompanied by an increase in LDH release (initial two days) followed by a decrease in LDH release or cytotoxic effects during an additional two days exposure to 0.3 μg/mL and 300 μg/mL erythromycin. The LDH activity measured in the supernatant reflects the stable cytosolic enzyme released upon cell lysis. For a five day experiment, the daily change in the media would result in the proliferation of cells. However, there is also spontaneous cell lysis which could reflect higher LDH. In addition, erythromycin is a known inhibitor of cell proliferation [38,39,40]. It may be the reason for lower levels of LDH during repeated treatment of erythromycin (chronic treatment). Compared to acute exposure, chronic exposure of erythromycin resulted in more substantial effects on the intestinal epithelium. The T84 cells grew faster when fresh medium was replaced daily and, therefore, the TER value of the cells without erythromycin kept increasing even after five days; however, the daily exposure of erythromycin to epithelial cells inhibited the growth and attachment of the cells.

Erythromycin acts as an inhibitor of intestinal sugar and amino acid (e.g., L-threonine and D-galactose) transportation and absorption [41,42,43]. Another macrolide class antimicrobial agent, rapamycin, slows down the proliferation of intestinal stem cells in the aging gut and induces autophagy in the intestinal epithelium [44]. Thus, we believe that the effect of erythromycin on the human intestinal epithelium observed in our study may be due to the role of erythromycin on suppressing the proliferation of intestinal cells.

Only one gene, *ICAM1,* encoding intercellular adhesion molecule 1 was significantly up-regulated in the single treatment with 0.3 μg/mL erythromycin. *ICAM1* and two other genes, *JAM3,* encoding junctional adhesion molecule 3, and *ITGA8,* encoding integrin alpha 8, were over-expressed in the single treatment with 300 μg/mL erythromycin. *ICAM1* is known to be involved in the formation of extracellular matrices [45]; thus, it is presumed that a higher concentration of erythromycin may damage the epithelial barrier, which could result in the passing of erythromycin into the basal media. The higher expression of *ICAM1* is a repair mechanism of the epithelial cells.

No significant changes in gene expression were observed after chronic-treatment with 0.3 μg/mL erythromycin. Only *ICAM2* was up-regulated after chronic treatment with 300 μg/mL erythromycin, but with less than a two-fold change (*p* < 0.01).

*ICAM1*, along with *ICAM2* and *JAM3*, play an important role in formation of extracellular matrices, as well as in tight junctions between epithelial cells. Tight junctions are crucial for the establishment and maintenance of cell polarity. An earlier study found that *ICAM1* can be up-regulated in the less differentiated cell line T84 by interferon-gamma and interleukin 1β [46]. This form of gene activation can be ruled out in this study, as the control cells did not show an increase in *ICAM1* gene expression. Tight junctions seal adjacent epithelial cells together, preventing the passage of most dissolved molecules as well as membrane-bound lipids and proteins between the apical and basolateral epithelial surfaces [47]. In the present study, the increased expression of these genes may be a mechanism by which epithelial cells are trying to protect themselves against the opening of the sealed tight junctions; however, integrity of the epithelial junction between cells depends on the coordinated interplay between several genes—a compensatory mechanism which in this study seems to be overcome by erythromycin treatment that leads to an increase in the TER. In an earlier report, in vivo studies showed that the involvement of *ICAM1* under pathologic conditions could increase accumulation of epithelial-associated neutrophils [48]. Moreover, epithelial *ICAM1* has an important role in the activation of epithelial Akt and β-catenin signaling, and neutrophils within the intestinal lumen are known to regulate epithelial homeostasis [49]. Even a subtle disruption of the ICAM-1/2 gradient may prevent migration of immune cells and inflammatory responses [50]. Considering the process of “egression” of immune cells at the intestinal epithelial layer, higher expression of *ICAM1* or *ICAM2* may initiate an inflammatory process during acute or chronic exposure to erythromycin. Another major component of tight junctions in epithelial cells are JAMs [51]. *JAM3* was identified as the counter-receptor of *JAM2* and leukocyte integrin Mac-1 [52,53]. It is also a component of desmosomes and a ligand for CD11b/CD18-mediated neutrophil trans-epithelial migration [54,55,56]. Previous studies highlighted an unexpected function for *JAM3* in controlling epithelial cell conversion from a static, polarized state to a pro-migratory phenotype [57,58]. It could be plausible that exposure to erythromycin may be changing the IEC polarization to a similar pro-migrating phenotype.

*ITGA8* was up-regulated in IECs during the acute exposure to erythromycin at the highest concentration. *ITGA8* plays an important role in focal adhesion. *ITGA8* is a receptor for fibronectin, vitronectin, tenascin-C fragments, osteopontin, and nephronectin [59,60,61], and has been shown to promote attachment of K562 cells and mesangial cells on fibronectin and vitronectin [59,62]. Previous studies suggested that *ITGA8* is important for maintaining integrity of the glomerular capillary tuft [63], regulating cell attachment and migration [62,64], regulating cell growth and survival [65,66], and attenuating proliferation and apoptosis [45,67,68]. The significantly higher up-regulation of *ITGA8* during acute exposure to high erythromycin dose clearly indicates that the IEC are trying to maintain homeostasis of the epithelial integrity by similar mechanisms. Moreover, cells exposed to the highest erythromycin concentrations showed same level of cytotoxicity as the residual level, which further confirms that the increased expression of *ITGA8* may be involved in the attenuation of the cell death. The acute exposure of erythromycin to IECs resulted in the up-regulation of genes involved in the function of tight junctions and focal adhesions, a possible indication that the epithelial cells were attempting to compensate for the increased permeability by enhanced expression of genes involved in maintaining cell–cell adhesion. The up-regulation of these three genes may also be related to the anti-inflammatory effects and immune-regulatory activity of erythromycin in human intestinal epithelial cells [69,70,71]. Thus, it could be postulated that the acute exposure of residual erythromycin may result in the induction of protective responses in healthy individuals.

Therapeutic doses of macrolide drugs (e.g., erythromycin, azithromycin, and roxithromycin) modulate immune responses in human monocyte-derived dendritic cells (DCs) and CD4+ T cells, suppress DC-induced allogeneic T-cell proliferation and cytokine production [69,70,71,72,73], and significantly decrease neutrophil and monocyte trans-endothelial migration [74]. Roxithromycin can also inhibit nuclear factor kappa-β signaling and endoplasmic reticulum stress in intestinal epithelial cells, and ameliorate experimental colitis in mice [75]. Erythromycin has a possible role in disrupting the signaling pathway that regulates nuclear factor-kappa-β activation [76,77,78]. The possibility of erythromycin to cause more adverse effects by conversion to AHE is beyond the scope of this study. Moreover, the expression of CYP3A4 by T84 cells is controversial [38,39,40]. Though it is beyond the scope of this current study, it would be very interesting to see the effects of residual doses of erythromycin in an animal model.

Apart from addressing the knowledge-gap regarding impact of residual erythromycin on intestinal permeability, this study also addresses a major lacuna in the area of transfer of antibiotic through breastmilk to infant and its relationship to intestinal health. Due to the low levels of erythromycin in breastmilk it is acceptable to use in nursing mothers [79,80]. Average erythromycin levels in the breastmilk after two hours of oral or intravenous exposure to nursing mothers were found to be 1 μg/mL and 2.5 μg/mL, respectively [81,82]. The lower amount of erythromycin in the breast milk of females that took oral medication may be due to the biotransformation of drug by intestinal bacteria [83]. Pyloric stenosis, vomiting, and poor weight gain, along with some other medical conditions were hypothesized to be related to erythromycin in breastmilk [84]. Our study also provides a research model to understand the mechanism of these intestinal disturbances if infants are exposed to erythromycin exposure via breast milk.

## 5. Conclusions

This study used a comprehensive approach to evaluate the effects of exposure to erythromycin on intestinal permeability, cytotoxicity, and expression of related genes in an in vitro model of the human intestinal epithelium. Exposure to erythromycin resulted in increased intestinal permeability at both the residue (0.3 μg/mL) and high-level (300 μg/mL) concentrations tested. The increase in intestinal permeability appeared to be positively correlated with cytotoxic effects after acute treatment, while negatively correlated with cytotoxicity effects after chronic treatment. The changes in intestinal permeability and cytotoxicity may be due to the role of erythromycin in suppressing the proliferation of intestinal cells. The exposure to erythromycin, especially at the higher concentration, could up-regulate genes associated with tight junctions and focal adhesions. This study opens the door for future challenging experiments using *in vivo*, *ex vivo*, or organ-on-a chip models of the human GIT to determine the potential adverse effects following chronic or long-term human exposure to antimicrobial residues in foods.

## Figures and Tables

**Table 1:** Differentially expressed genes after 48 h single treatment by erythromycin. (0.3 and 300 μg/mL).

Single Exposure for 48 h					
Gene	Function	Ery 0.3/Ery 0	Ery 0.3/Ery 0	Ery 300/Ery 0	Ery 300/Ery0
		Fold Change	*p* Value	Fold Change	*p* Value
**Tight junction**					
*ICAM1*	Intercellular adhesion molecule 1	2.37	0.000636	2.05	0.016607
*JAM3*	Junctional adhesion molecule 3	2.13	0.062148	2.65	0.040630
*CLDN10*	Claudin 10	–7.4	0.373085	–5	*0.378114*
*CLDN11*	Claudin 11	3.68	0.944793	2.85	0.999572
*CLDN14*	Claudin 14	–1.55	0.592628	3.83	0.299305
*CLDN17*	Claudin 17	–2.74	0.452451	6.44	0.369806
*CLDN18*	Claudin 18	2.02	0.402447	–1.57	0.633391
*CLDN19*	Claudin 19	–2.32	0.728604	–3.51	0.727239
*CLDN6*	Claudin 6	1.27	0.465959	2.22	0.264438
**Focal adhesion**					
*CAV1*	Caveolin 1, caveolae protein	–2.94	0.164653	–1.32	0.390713
*CAV3*	Caveolin 3	14.52	0.120646	1.15	0.556335
*ITGA4*	Integrin, alpha 4	5.07	0.385157	–1.13	0.841795
*ITGA7*	Integrin, alpha 7	1.65	0.618012	2.2	0.367128
*ITGA8*	Integrin, alpha 8	2.76	0.382339	175.02	0.024344
*ITGAM*	Integrin, alpha M	–1.06	0.902768	3.21	0.308094
**Gap junction**					
*GJA1*	Gap junction protein, alpha 1	–1.2	0.795080	–5.32	0.055363
*GJA3*	Gap junction protein, alpha 3	–1.33	0.417673	3.53	0.368254
*GJA5*	Gap junction protein, alpha 5	2.93	0.364299	12.55	0.216151
*GJB4*	Gap junction protein, beta 4	1.65	0.342500	2.01	0.286874
*GJB5*	Gap junction protein, beta 5	1.32	0.365400	2.28	0.286658
*GJB6*	Gap junction protein, beta 6	–1.28	0.092314	1.67	0.420076
*GJC3*	Gap junction protein, epsilon 1	1.95	0.367178	–25.08	0.263848
**Adherens junction and desmosomes genes**					
*DSC1*	Desmocollin 1	–46.06	0.144780	–31.11	0.146747
*DSG3*	Desmoglein 3	2.18	0.307182	3	0.088211
*CDH2*	Cadherin 2, type 1, N-cadherin	–1.28	0.092314	4.81	0.336937
*NOTCH4*	Notch 4	1.71	0.217780	2.75	0.225017
*PVRL1*	Poliovirus receptor-related 1	1.42	0.011171	1.57	0.264911

**Table 2:** The differentially expressed genes after 5 days multi-treatment by erythromycin (0.3 μg/mL and 300 μg/mL).

Multi Treatment for 5 Days					
Gene	Function	Ery 0.3/Ery 0	Ery 0.3/Ery 0	Ery 300/Ery 0	Ery 300/Ery 0
		Fold Change	*p* Value	Fold Change	*p* Value
**Tight junction**					
*ICAM2*	Intercellular adhesion molecule 2	–1.09	0.308601	1.2	0.000850
*CLDN10*	Claudin 10	–1.09	0.649218	4.34	0.364360
*CLDN14*	Claudin 14	–14.64	0.121784	–2.79	0.217719
*CLDN17*	Claudin 17	–2.14	0.392266	–2.6	0.371088
*CLDN18*	Claudin 18	3.18	0.298743	8.38	0.132072
*CLDN19*	Claudin 19	8.2	0.099657	1.41	0.596306
*CLDN8*	Claudin 8	–2.05	0.235259	1.97	0.191047
**Focal adhesion**					
*CAV3*	Caveolin 3	–14.02	0.158030	–3.76	0.284148
*ITGA4*	Integrin, alpha 4	–3.86	0.372310	–3.91	0.367691
*ITGAM*	Integrin, alpha M	–3.77	0.596918	1.22	0.803690
*ITGB3*	Integrin, beta 3	–2.93	0.201419	–1.26	0.410330
**Adherens junction and desmosomes genes**					
*DSC1*	Desmocollin 1	–1.07	0.969266	–2.69	0.136576
*DSG3*	Desmoglein 3	8.14	0.293908	9.94	0.165064
**Gap junction**					
*GJA5*	Gap junction protein, alpha 5	1.35	0.454641	6.77	0.330339
*GJB6*	Gap junction protein, beta 6	4.39	0.375203	1.12	0.596642
*GJC2*	Gap junction protein, gamma 2	1.204	0.78586	–2.78	0.688153
